# Prevalence and association of human papillomavirus, Epstein-Barr virus and Merkel Cell polyomavirus with neoplastic esophageal lesions in northern Iran

**DOI:** 10.22088/cjim.9.4.353

**Published:** 2018

**Authors:** Yousef Yahyapour, Rabeae Rahmani, Majid Alipour, Ahad Alizadeh, Aynaz Khademian, Farzin Sadeghi

**Affiliations:** 1 Infectious Diseases and Tropical Medicine Research Center, Babol University of Medical Sciences, Babol, Iran; 2 Department of Microbiology, Babol University of Medical Sciences, School of Medicine, Babol, Iran; 3 Department of Biology, Azad University of Babol, Babol, Iran; 4 Department of Epidemiology and Reproductive Health, Reproductive Epidemiology Research Center, Royan Institute for Reproductive Biomedicine, ACECR, Tehran, Iran; 5 Cellular and Molecular Biology Research Center, Health Research Institute, Babol University of Medical Sciences, Babol, Iran

**Keywords:** Esophageal squamous cell carcinoma, Human papillomavirus, Epstein-Barr virus, Merkel cell polyomavirus

## Abstract

**Background:**

Studies concerning on esophageal squamous cell carcinoma (ESCC) etiological factors have been done for several decades, however, results reported from various investigations were not consistent. The present investigation aimed to explore the presence of 3 oncogenic viruses, human papilloma virus (HPV), Epstein-Barr virus (EBV) and Merkel cell polyomavirus (MCPyV) in the neoplastic and non- neoplastic esophageal lesions collected from Mazandaran, a high risk area of Iran.

**Methods:**

In total, 168 esophageal specimens (100 with ESCC confirmed diagnosis and 68 without esophageal malignancy) were analyzed for HPV, EBV and MCPyV by Real Time PCR.

**Results:**

HPV DNA was detected in 27 out of the 100 neoplastic esophageal lesions (27.0%) and 28 out of the 68 samples from non-neoplastic group (41.2%). EBV DNA was detected in esophageal specimens of 10 out of the 100 neoplastic cases (10%) and 3 out of the 68 samples in non- neoplastic group (4.4%). MCPyV DNA was detected in esophageal specimens of 30 out of the 100 neoplastic cases (30.0%) and 24 out of the 68 samples in non- neoplastic group (35.3%). There was no statistically significant difference in HPV (p=0.066), EBV (p=0.143) and MCPyV (p=0.471) DNA positivity between neoplastic and non-neoplastic groups.

**Conclusions:**

This study showed that HPV, EBV and MCPyV can be detected in both neoplastic and non-neoplastic esophageal tissues and weakens the hypothesis of the pathogenic role of these viruses in esophageal malignant transformation.

Esophageal cancer is one of the most common causes of cancer death worldwide, with a very poor survival rate (1, 2). The incidence rate is high in certain parts of Asia, including China and northern Iran, wherein esophageal squamous cell carcinoma (ESCC) is predominant (3, 4). The main etiological factors for the development of ESCC have not been completely understood. Alcohol drinking, tobacco smoking and dietary or environmental factors was thought to play a role in the carcinogenesis (5, 6). In recent years, accumulating evidence has suggested that infection with oncogenic viruses may have pathogenetic significance in the development of ESCC (7, 8). Among these viruses, the core issue was focused on the infection of human papillomavirus (HPV), but causal relationship between HPV infection and risk of ESCC remained disputable (9-12). In addition, possible etiologic roles of other oncogenic viruses either alone or together with HPV in the development of ESCC are suggested (7, 8).

Contribution of Epstein-Barr virus (EBV) has been demonstrated in several epithelial malignancies, including nasopharyngeal carcinoma (NPC) and gastric carcinoma (13). The esophagus has anatomic vicinity with nasopharynx and stomach, and esophageal cancer has histological similarities to NPC (14). Recently, some investigators have proposed an association between EBV and ESCC, but their results are still controversial (15-19). Merkel cell polyomavirus (MCPyV), the most recently discovered human tumor virus has been accepted as the etiological factor in the majority of Merkel cell carcinoma (MCC), a rare and highly aggressive skin cancer (20). In spite of the fact that the route of MCPyV transmission has not been determined, MCPyV genome fragments have been demonstrated in the upper aerodigestive tract including esophagus (21, 22). If the upper aerodigestive tract is exposed constantly to this tumorigenic virus, it is conceivable that malignancy may develop in this region. Hence, to investigate whether these 3 oncogenic viruses could have a role in the etiology of ESCC, the prevalence of infection with aforementioned viruses were explored in the neoplastic and non- neoplastic esophageal lesions collected from Mazandaran, which is one of the highest ESCC risk areas in Iran.

## Methods

**Patients and Tissue Specimens: **The present case control study included 168 formalin-fixed paraffin-embedded resection specimens from esophagus that were collected from the archives of two referral pathology centers affiliated to Babol University of Medical Sciences in Mazandaran, northern Iran. One-hundred out of 168 specimens had ESCC with confirmed diagnosis and 68 specimens diagnosed without malignant neoplasm in esophagus. The samples with ESCC histopathologic diagnosis were classified according to tumor differentiation grade (23). None of the subjects had received immunosuppressive therapy prior to endoscopy or surgery. This study was approved by the Ethics Committee of Babol University of Medical Sciences (Mubabol.REC.1394.79), and for all subjects, written informed consent was obtained.

**DNA Extraction: **Five µm thick tissue sections were de-waxed according to a previously described procedure (24). DNA was extracted from each tissue sample, using the High Pure PCR Template Preparation Kit (Roche Diagnostics, Mannheim, Germany) according to the manufacturer’s instructions. The quality and quantity of purified DNA was determined using a NanoDrop spectrophotometer (Thermo Scientific, Wilmington, USA). In addition, DNA integrity in each tissue sample was evaluated using human RNase P gene (RPP30) amplification based on a previously described procedure (25). Sterile microcentrifuge tubes containing only reaction mixtures were processed simultaneously with the tissue samples as a DNA isolation negative control.

**HPV** **DNA Detection and Genotyping: **HPV DNA detection was performed using the qualitative Real Time PCR with L1 consensus primers (MY09 and MY11) as described previously (26). Viral genotyping was carried out in HPV-DNA positive samples using the AmpliSense HPV real-time fluorescence detection (FRT) kit (Central Research Institute of Epidemiology, Moscow, Russia) following instruction manual. This assay can reliably detect 15 different genotypes of HPV, including types 16, 18, 31, 33, 35, 39, 45, 51, 52, 56, 58, 59 as high-risk genotypes and types 6 and 11 as low-risk genotypes.

**EBV DNA Detection: **The presence of EBV DNA sequences was investigated by qualitative real-time PCR using an ABI 7300 Real-Time PCR System (Applied Biosystems, Branchburg, NJ, USA) with the primer sets and TaqMan probe speciﬁc for the EBER gene of EBV as described elsewhere (27). In brief, 500 ng of extracted DNA was added to PCR mixture containing 12.5 µl YTA 2X Multiplex Real-Time PCR Smart mix (Yekta Tajhiz Azma, Tehran, Iran), 0.3 µM each primer and 0.2 µM dual-labeled probe. DNA extracted from supernatant of EBV-producing B-cell line (B95-8) was used as a positive control (28). Each real-time PCR run included reaction mixtures without DNA template as a negative control. Before testing the clinical samples, the specificity of the real-time PCR technique was evaluated using positive and negative control samples.

**MCPyV DNA Detection and Quantitation: **Real-time PCR method was performed to detect and quantify the amount of MCPyV DNA load as the viral DNA copies per cell according to a previously described procedure (29). Preparation of real-time PCR calibration standards (plasmids containing cloned target sequences of MCPyV and human RNase P gene) was described elsewhere (30). The normalized value of the MCPyV DNA load was calculated by dividing the virus copy number by half of the RNase P gene copy number, because each diploid cell contains two copies of RNase P gene.

**Statistical Analysis: **Statistical analyses were done by arm and base packages of R software, version 3.2.4. Normality of quantitative variables was checked by Shapiro-Wilks test. Quantitative variables were reported using 95% credible interval or mean ± standard deviation (SD). The differences between normal variables were compared by independent t test or one way analysis of variance (ANOVA). The differences between nonparametric variables were compared by Mann–Whitney U or Kruskal–Wallis tests. Regarding the small number of samples, Bayesian logistic regression was used to calculate adjusted and crude odds ratios (ORs) and 95 % credible intervals (CI) using MCMC algorithm. A p-value of ≤0.05 was considered to be statistically significant. 

## Results

**Demographic and Baseline Characteristics: **The current cross-sectional study investigated 168 esophageal lesions. According to the histopathologic diagnosis, study participants were divided in two groups: one-hundred subjects had neoplastic esophageal lesions, with ESCC confirmed diagnosis (male 59, female 41) (mean age, 66.8±10.7 years; range, 38-91 years) and 68 subjects had no neoplasia in esophagus (male 42, female 26) (mean age, 64.2±12.5 years; range, 42-90 years). There was no statistically significant difference between age (P=0.151) and gender (P=0.719) in neoplastic and non- neoplastic groups. Considering urban/rural residence, out of 100 subjects in neoplastic group 48 (48%) were urban and 52 (52%) were rural. From the 68 subjects in non-neoplastic group, 37 (54.4%) and 31 (45.6%) lived in urban and rural areas, respectively. The samples with ESCC histopathologic diagnosis were classified according to tumor differentiation grade (23) and as follows: out of the 100 samples, 64 (64.0%) were classified as not differentiated, 3 (3.0%) poorly differentiated, 16 (16.0%) moderately differentiated and 17 (17.0%) well differentiated tumor samples. The non-neoplastic esophageal samples were categorized based on histopathologic criteria: 9 (13.2%) samples were diagnosed with esophageal dysplasia, 52 (76.5%) with esophagitis and 7 (10.3%) had normal histology. In terms of location of esophageal lesions, in neoplastic group the most common location was the middle third of esophagus with 41 (41%) cases, followed by the lower third and upper third with 35 (35%) and 24 (24%) cases, respectively. Out of the 68 non-neoplastic esophageal specimens, 34 (50%) cases were located at the lower third of esophagus, followed by 12 (17.6%) cases and 9 (13.2%) cases in the upper third, and middle third respectively. The location of esophageal specimen was unknown in 13 (19.1 %) subjects in non-neoplastic group.

**Detection of HPV and Viral Genotypes in Neoplastic and Non-neoplastic Lesions: **Totally, HPV DNA sequences were detected in 55 (32.7%) out of the 168 tested samples, of which 15 (27.3 %) were infected with a single HPV type and six (10.9%) with two or more HPV types (multiple-type infection). Human papillomavirus genotype was not recognized in 34 (61.8%) positive samples (untypable). Specifically, HPV DNA was detected in 27 out of the 100 neoplastic esophageal lesions (27.0%). Based on tumor differentiation grade, 34.4% of not differentiated, 6.3% of moderately differentiated and 23.5% of well differentiated tumors were HPV DNA positive. In addition, HPV DNA was detected in 28 out of the 68 samples from non-neoplastic group (41.2%). According to histopathologic diagnosis, 33.3% of esophageal dysplasia, 46.2% of esophagitis, and 14.3% of samples with normal histology were HPV DNA positive. There was no statistically significant difference in HPV DNA positivity between neoplastic and non-neoplastic groups (P=0.066). Among the neoplastic esophageal lesions, 25.9% (7.27) harbored high risk types (HPV35, HPV39 and HPV45 were the most common high-risk types in neoplastic group, which were detected in 3 cases each), 18.5% (5.27) harbored low risk types and 62.9% (17.27) were untypable. In non-neoplastic group 21.4% (6.28) harbored high risk types (HPV56 was the most common high-risk type in non-neoplastic group, which was detected in 2 cases), 17.8% (5.28) harbored low risk types and 60.7% (17.28) were untypable. Multiple-type infection was seen in 22.2% (6.27) of neoplastic esophageal lesions, but in none of the 28 samples from non- neoplastic group (table 1). Regarding the location of esophageal lesions in neoplastic group, 20.8% (5.24) of the upper third, 36.6% (15.41) of the middle third, and 20.0% (7.35) of the lower third specimens were HPV DNA positive. In non-neoplastic esophageal lesions, 8.3% (1.12) of upper third, 55.6% (5.9) of the middle third, 61.8% (21.34) of the lower third, and 7.7% (1.13) of the unknown third specimens were HPV DNA positive.

**Detection of EBV in Neoplastic and Non-neoplastic Lesions: **Of the 168 tested samples, EBV DNA sequence was found in 13 (7.7%). EBV DNA was detected in esophageal specimens of 10 out of the 100 (10%) neoplastic cases and 3 out of the 68 samples in non- neoplastic group (4.4%). There was no statistically significant difference in EBV DNA positivity between neoplastic and non-neoplastic groups (P=0.143). In detail, EBV DNA was detected in12.5% (8.64) of not differentiated, and 11.8% (2.17) of well differentiated, but in none of the poorly differentiated and moderately differentiated tumors. In non- neoplastic group, only 5.8% (3.52) of esophagitis specimens contained EBV DNA sequence and EBV infection was not found in samples with esophageal dysplasia and normal histology. Based on the location of esophageal lesions in neoplastic group, 8.3% (2.24) of the upper third, 7.3% (3.41) of the middle third, and 14.3% (5.35) of the lower third specimens were EBV DNA positive. In non-neoplastic esophageal lesions, 5.9% (2.34) of the lower third, and 7.7% (1.13) of the unknown third specimens were EBV DNA positive.

**Table 1:** Frequency of HPV infection in patients with neoplastic and non-neoplastic esophageal lesions

	**Study group**		**OR (95% CI)** *	**P value**
	**Neoplastic Esophageal ** **Lesions (n=100)**	**Non-Neoplastic Esophageal** **Lesions (n=68)**		
Number HPV positive(% of all)	27 (27.0%)	28 (41.2%)	0.53 (0.261,1.072)	0.066
Number positive for HPV high-risk types(% of HPV positive)	7.27 (25.9%)	6.28 (21.4 %)	0.777 (0.249,2425)	0.771
Number positive for HPV low-risk types(% of HPV positive)	5.27 (18.5 %)	5.28 (17.8 %)	0.665 (0.146,3.016)	0.528
Single-type infection(% of HPV positive)	4.27 (14.8%)	11.28 (39.3%)	0.218 (0.048,0.779)	0.011
Multiple-type infection(% of HPV positive)	6/27 (22.2 %)	0 (0%)	-	0.082
Untypable(% of HPV positive)	17.27 (62.9%)	17.28 (60.7%)	0.616 (0.269,1.411)	0.242

* Crude odds ratio

**Detection and Quantitation of MCPyV in Neoplastic and Non-neoplastic Lesions: **In the present study, neoplastic and non-neoplastic esophageal specimens were examined for the presence of MCPyV sequence by quantitative real-time PCR. Out of the 168 tested samples, the MCPyV DNA sequence was found in 54 (32.1%). MCPyV DNA was detected in esophageal specimens of 30 out of the 100 neoplastic cases (30.0%) and 24 out of the 68 samples in non- neoplastic group (35.3%). There was no statistically significant difference in MCPyV DNA positivity between neoplastic and non-neoplastic groups (P=0.471). Regarding the grade of tumor in neoplastic group, MCPyV infection was detected in 31.3% (20/64) of not differentiated, 33.3% (1.3) of poorly differentiated, 18.8% (3.16) of moderately differentiated, and 35.3% (6.17) of well differentiated tumors. In non-neoplastic group, 55.6% (5.9) of esophageal dysplasia, and 36.5% (19.52) of esophagitis specimens contained MCPyV DNA sequence. Merkel cell polyomavirus DNA sequence was not found in samples with normal histology. According to the location of esophageal lesions in neoplastic group, 29.2% (7.24) of the upper third, 24.4% (10.41) of the middle third, and 37.1% (13.35) of the lower third specimens were MCPyV DNA positive. In non-neoplastic esophageal lesions, 41.7% (5.12) of the upper third, 22.2% (2/9) of the middle third, 35.3% (12/34) of lower third, and 38.5% (5.13) of the unknown third specimens were MCPyV DNA positive. 

The mean MCPyV DNA load was 5.4×10^-6^±13.2×10^-6^ and 5.6×10^-^ ±17.8×10^-6^ per cell in neoplastic cases and non-neoplastic samples, respectively.

**Figure 1:**
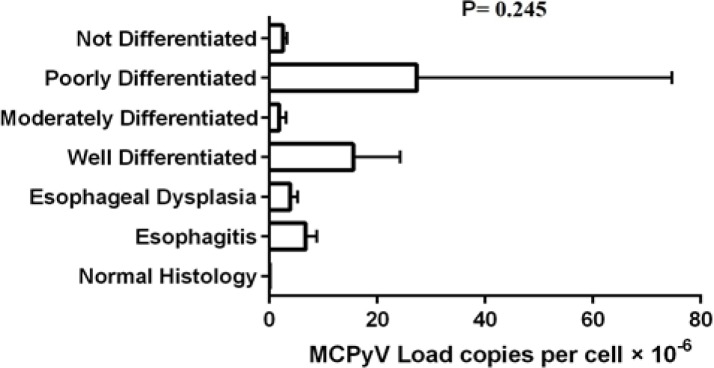
The mean MCPyV DNA load according to histopathologic diagnosis in patients with neoplastic and non-neoplastic esophageal lesions. The p-value was determined by the KruskalWallis test

There was no statistically significant difference between neoplastic cases and non-neoplastic samples regarding mean MCPyV DNA load (p=0.397). Additionally, the mean MCPyV copy number was higher in poorly differentiated (27.3×10^-6^±47.3×10^-6^) and well differentiated (15.5×10^-6^±35.8×10^-6^) tumors compared to other histopathologic groups however, this difference was not statistically significant (p=0.245) (fig. 1).

**Concomitant Viral Infections** **in Neoplastic and Non-neoplastic Lesions: **In total, concomitant double and triple infection with HPV, EBV and MCPyV was seen in 16(16.0%) neoplastic cases and 17(25.0%) non-neoplastic esophageal samples. No statistically significant difference was found between neoplastic and non-neoplastic samples regarding concomitant double and triple viral infection (table 2).

**Association between Viral Infections and ESCC Development: **According to Bayesian logistic regression analysis, a significant inverse association between HPV infection and odds of ESCC was observed (OR, 0.36; 95 % CI: 0.17-0.77). Although EBV infection was related to higher risk of ESCC, it did not get the significant level (OR, 3.21; 95 % CI: 0.75-13.57). 

Furthermore, MCPyV infection was also inversely associated with odds of ESCC, though the association did not reach a significant level (OR, 0.97; 95 % CI: 0.46-2.08). Adjustment for age, gender and infection with aforementioned viruses in Bayesian logistic regression model increased odds of ESCC in the middle third of esophagus (OR, 3.01; 95 % CI: 1.08-8.33) (table 3).

**Table 2:** Frequency of concomitant double and triple infection with HPV, EBV and MCPyV in patients with neoplastic and non-neoplastic esophageal lesions

	**Study group**			**OR (95% CI)** *	**P-value**
	**Neoplastic Esophageal Lesions (n=100)**	**Non-Neoplastic Esophageal Lesions (n=68)**	**Total** **(n=168)**		
HPV and EBV positive (%)	2 (2%)	2 (2.9%)	4 (2.4%)	0.675 (0.048,9.528)	1
HPV and MCPyV positive (%)	11 (11%)	11 (16.2%)	22 (13.1%)	0.642 (0.235,1.753)	0.358
EBV and MCPyV positive (%)	3 (3%)	2 (2.9%)	5 (3%)	1.02 (0.114,12.533)	1
HPV and EBV and MCPyV positive (%)	0 (0%)	2 (2.9%)	2 (1.2%)	-	0.056

* Crude odds ratio

**Table 3:** Bayesian logistic regression analysis of the association between location of esophageal specimen, HPV, EBV and MCPyV infection and ESCC

**Parameter**	**Odds Ratio (OR) of ESCC ** £ (95% CI)**	**P-value**
HPV infection	0.36 (0.17-0.77)	**0.007**
EBV infection	3.21 (0.75-13.57)	0.111
MCPyV infection	0.97 (0.46-2.08)	0.959
Location of esophageal specimen* Middle third Lower third	3.01 (1.08-8.33)	0.032
	0.64 (0.27-1.49)	0.299

* Upper third of the esophagus is taken as a reference category

** The results were adjusted by age, gender and MCPyV DNA load

£ ESCC: Esophageal squamous cell carcinoma

## Discussion

Incidence rates, histological subtypes, and etiological factors for esophageal cancer differ considerably between Asian and Western countries (1). Northern Iran, with markedly high ESCC incidence rate is located in the so-called “Asian esophageal cancer belt”(3). Reasons for high ESCC occurrence in this region are still unclear. Infection with oncogenic viruses as a contributor to ESCC has been hypothesized by several investigators (7, 8). HPV, EBV and MCPyV are well known oncogenic viruses associated with the development of different malignancies. To investigate whether these 3 oncogenic viruses could have a role in the etiology of ESCC in northern Iran, a cross-sectional study was designed and a total of 168 neoplastic and non-neoplastic esophageal specimens were examined for HPV, EBV and MCPyV infections.

 In the present study, both neoplastic and non-neoplastic esophageal specimens were found to harbor HPV DNA. However, HPV infection in non-neoplastic lesions (41.2%) was considerably higher than ESCC cases (27.0%). The higher rate of HPV infection in our non-neoplastic group might be explained by histopathologic distribution of samples (76.5% of non-neoplastic samples were diagnosed with esophagitis and only 10.3% had normal histology). HPV infection was frequently detected in esophagitis samples by other investigators (31, 32). 

Genotype characterization for HPV revealed 12 different types, including 10 high-risk types and 2 low-risk types. HPV35, HPV39 and HPV45 were the most common high-risk types in neoplastic group, while HPV56 was most prevalent high-risk type in non-neoplastic samples. Infection with HPV18 was not found in both neoplastic and non-neoplastic samples; only 1 ESCC specimen with HPV16 genotype was recognized. The results of HPV genotyping in the current study are in contrast with a number of reports, which identified HPV16 and HPV18 as the two most common HPV high-risk types in ESCC (32-34). 

In the present study, HPV infections caused by unknown or untypable genotypes were found in high rates in both neoplastic (62.9%) and non-neoplastic (60.7%) groups, which might be explained by a broad spectrum of different HPV genotypes that was not identified by our HPV genotyping kit. These data are consistent with a number of reports, which identified diverse and putative new HPV genotypes in cancerous and non-cancerous esophageal specimens (31, 35, 36).

In the current investigation, EBV DNA was found in 10% of neoplastic and 4.4% of non-neoplastic esophageal specimens and EBV infection was not found in samples with normal histology. Although EBV infection was related to higher risk of ESCC, but the result was not statistically significant. It has been demonstrated that, there is a higher rate of EBV- associated ESCC in Asian countries than in Western countries (7, 19, 37, 38). 

The different results of EBV-associated ESCC from the different regions could be affected by the racial and geographical variables (16, 39, 40). In good concordance with our previous report, a low copy number of MCPyV DNA was detected in both neoplastic and non-neoplastic esophageal specimens (41). The difference between MCPyV DNA loads (as a copy per cell) in neoplastic and non-neoplastic groups was not statistically significant. Low copy numbers of MCPyV genome in both cancerous and non-cancerous esophageal tissues might be explained by simple persistent viral replication in esophagus as a passenger virus or viral shedding from another organ (e.g. respiratory tract) to esophagus without any pathological outcome. Generally, if MCPyV-prompted tumor in humans arose as a monoclonal expansion of a virally transformed cell (e.g., MCC tumors); more than one DNA copy per tumor cell would be present (20). In comparison with previous publications in our region, present investigation was done in a larger variety of neoplastic and non-neoplastic esophageal specimens and to our knowledge, this is first study in the EMRO region to investigate the role of these three oncogenic viruses (HPV, EBV& MCPyV) in ESCC using highly sensitive real-time PCR technique. The results of the current investigation should be interpreted cautiously, due to some limitations including, the small percentage of samples with normal histology as a control and lack of fresh biopsy specimens for analysis. Assessment of fresh biopsy specimens by quantitative real-time PCR technique may shed more light on the role of aforementioned oncogenic viruses in the development of esophageal cancer. 

In conclusion, the present study showed that HPV, EBV and MCPyV can be detected in both neoplastic and non-neoplastic esophageal tissues. The findings of the current study weaken the hypothesis of the pathogenic role of aforementioned oncogenic viruses in esophageal malignant transformation. Yet, to ascertain our results, more world-wide epidemiological studies with larger sample size and preferably on fresh biopsy specimens should be done. 
